# Factors related to large bone defects of bipolar lesions and a high number of instability episodes with anterior glenohumeral instability

**DOI:** 10.1186/s13018-021-02395-5

**Published:** 2021-04-13

**Authors:** Noboru Matsumura, Kazuya Kaneda, Satoshi Oki, Hiroo Kimura, Taku Suzuki, Takuji Iwamoto, Morio Matsumoto, Masaya Nakamura, Takeo Nagura

**Affiliations:** 1 Department of Orthopedic Surgery, Keio University School of Medicine, 35 Shinanomachi, Shinjuku-ku, Tokyo, 160-8582 Japan; 2 Department of Clinical Biomechanics, Keio University School of Medicine, 35 Shinanomachi, Shinjuku-ku, Tokyo, 160-8582 Japan

**Keywords:** Glenohumeral instability, Glenoid defect, Hill-Sachs lesion, Humeral head defect, Bipolar lesion, Shoulder dislocation, Glenohumeral dislocation

## Abstract

**Background:**

Significant bone defects are associated with poor clinical results after surgical stabilization in cases of glenohumeral instability. Although multiple factors are thought to adversely affect enlargement of bipolar bone loss and increased shoulder instability, these factors have not been sufficiently evaluated. The purpose of this study was to identify the factors related to greater bone defects and a higher number of instability episodes in patients with glenohumeral instability.

**Methods:**

A total of 120 consecutive patients with symptomatic unilateral instability of the glenohumeral joint were retrospectively reviewed. Three-dimensional surface-rendered/registered models of bilateral glenoids and proximal humeri from computed tomography data were matched by software, and the volumes of bone defects identified in the glenoid and humeral head were assessed. After relationships between objective variables and explanatory variables were evaluated using bivariate analyses, factors related to large bone defects in the glenoid and humeral head and a high number of total instability episodes and self-irreducible dislocations greater than the respective 75th percentiles were evaluated using logistic regression analyses with significant variables on bivariate analyses.

**Results:**

Larger humeral head defects (*P* < .001) and a higher number of total instability episodes (*P* = .032) were found to be factors related to large glenoid defects. On the other hand, male sex (*P* = .014), larger glenoid defects (*P* = .015), and larger number of self-irreducible dislocations (*P* = .027) were related to large humeral head bone defects. An increased number of total instability episodes was related to longer symptom duration (*P* = .001) and larger glenoid defects (*P* = .002), and an increased number of self-irreducible dislocations was related to larger humeral head defects (*P* = .007).

**Conclusions:**

Whereas this study showed that bipolar lesions affect the amount of bone defects reciprocally, factors related to greater bone defects differed between the glenoid and the humeral head. Glenoid defects were related to the number of total instability episodes, whereas humeral head defects were related to the number of self-irreducible dislocations.

## Introduction

Glenohumeral instability is a common pathology in young people [1], and good clinical results can be expected by stabilizing surgery [2]. However, recurrence occurs often after stabilization, and significant bone defects in the glenoid are associated with failed surgical stabilization [3–8] and poor clinical outcomes [9]. Although most papers about bone defects related to glenohumeral instability have focused on glenoid bone defects, a Hill-Sachs lesion, a posterolateral humeral head compression fracture caused by impact with the glenoid rim during an instability event [10], has also been recognized as a factor affecting instability in recent years [3, 5, 6, 8]. The glenoid track concept [11], in which the humeral head overrides the glenoid rim when the Hill-Sachs lesion extends more medially than the glenoid track, is now widely accepted, and bipolar bone loss is thought to affect shoulder instability reciprocally [11–13]. Since bone defects in the glenoid and in the humeral head adversely affect the clinical results after surgical stabilization [3–6], it is important to clarify the factors related to large bone defects in cases with glenohumeral instability.

In most cases with traumatic glenohumeral instability, repetitive traumatic episodes of the glenohumeral joint occur [14–17]. Increased instability episodes can impair the quality of life and activities of the patient. Some instability episodes cannot be reduced by themselves, which is a so-called dislocation, whereas an obvious event for which manual reduction was not required is a so-called subluxation [5, 14, 16, 18, 19]. Owens et al. [19] demonstrated that 85% of traumatic instability episodes are glenohumeral subluxations rather than dislocations. However, in which cases the number of instability episodes increases is still unclear, and little is known about the difference in instability episode type.

Although multiple factors are supposed to affect the creation and enlargement of bone defects [20] and an increased number of instability episodes, the factors related to bone defects and instability episodes have not been sufficiently evaluated. Few studies [13, 21, 22] have evaluated both glenoid bone and humeral head defects in the same shoulders. The purpose of this study was to identify factors related to large bone defects and a high number of instability episodes in patients with glenohumeral instability. We hypothesized that there is a relationship among the size of bone defects, the number of traumatic episodes, and patients’ characteristics and that factors related to large bone defects differ between bipolar bone lesions.

## Methods

### Patients

This was a retrospective, observational study of 164 consecutive cases with anterior glenohumeral instability whose bone defects of the glenoid and humeral head were evaluated preoperatively by computed tomography (CT) during the period between 2010 and 2018. The inclusion criteria for this study were shoulders with symptomatic anterior instability of the glenohumeral joint with traumatic episodes and shoulders with unilateral instability. The exclusion criteria were cases of bilateral glenohumeral instability (35 cases), previous shoulder stabilization surgery (4 cases), glenohumeral arthritis on CT (2 cases), and full-thickness rotator cuff tear detected during stabilization surgery (3 cases). Thus, a total of 120 patients (96 males and 24 females; mean age, 26.1 ± 10.4 years; age range, 15–67 years) with symptomatic unilateral instability of the glenohumeral joint were reviewed. The mean age at the time of initial trauma was 19.7 ± 5.5 years (range, 10–41 years), with mean symptom duration of 6.4 ± 9.1 years (range, 0.1–53.0 years). The dominant shoulder was involved in 72 cases, and the nondominant one was involved in 48 cases. Arthroscopic Bankart repair was performed in 75 cases, open Bankart repair was performed in 49 cases, and open Latarjet reconstruction was performed in 6 cases. Bankart lesions in the anteroinferior labrum or bony Bankart lesions in the anteroinferior glenoid rim were found in all cases during stabilization surgery.

### Quantitative assessment of bone defects

**Fig. 1:**
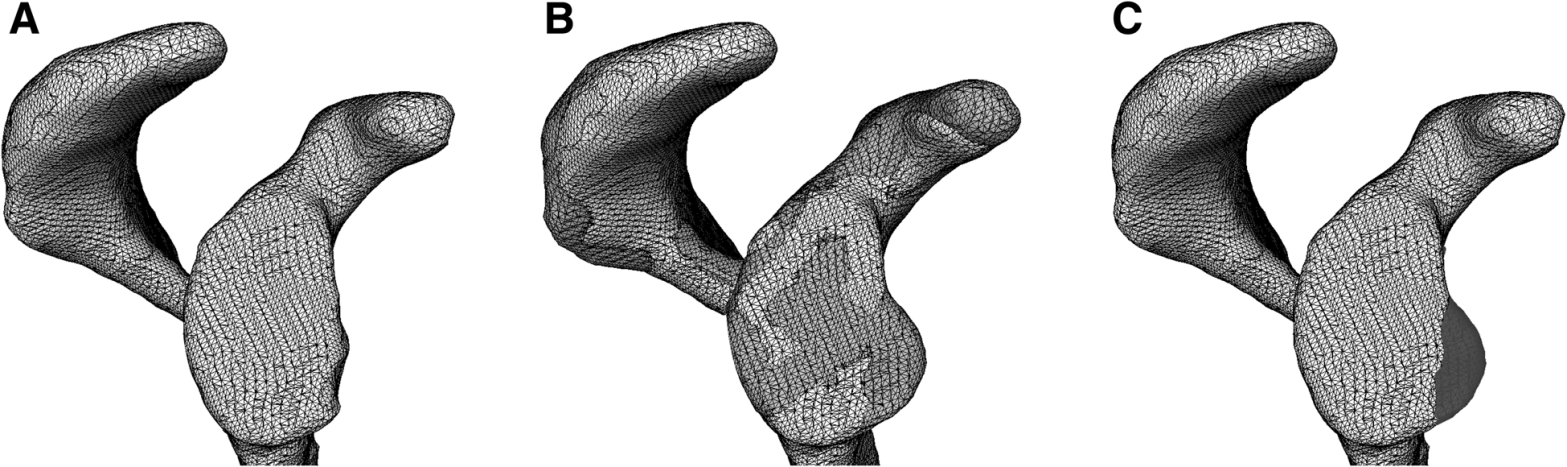
Identification of glenoid bone defects. **a** Three-dimensional surface model of the right glenoid (involved side) is reconstructed from the obtained computed tomography data. **b** The mirrored surface data of the left glenoid (intact side) is matched to the right glenoid model using the posterior half of the glenoid. **c** The bone defect area (black) is identified with subtraction of the affected-side volume from the intact side

**Fig. 2:**
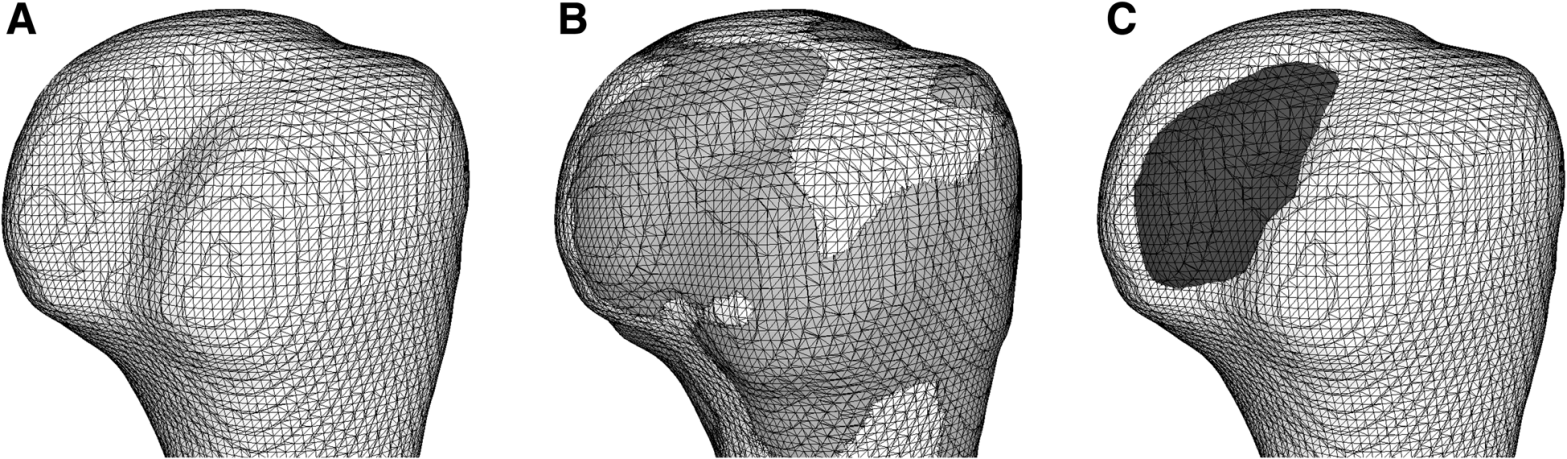
Identification of humeral head bone defects. **a** Three-dimensional surface model of the right proximal humerus (involved side) is reconstructed from the obtained computed tomography data. **b** The mirrored surface data of the left proximal humerus (intact side) is matched to the right glenoid model using the anterior half of the proximal humerus. **c** The bone defect area (black) is identified with subtraction of the affected-side volume from the intact side

To assess the amount of bone defects accurately, the present study three-dimensionally evaluated the bone defect volume in the glenoid and the humeral head referring to the contralateral intact shoulders. Since the dose in the CT gantry and the elapsed time did not change between unilateral and bilateral shoulder scanning, bilateral scanning was performed to detect the presence and amount of bone defects in cases with glenohumeral instability. Bone defect volume was calculated by a three-dimensional surface registration technique referring to the contralateral intact shoulder, and its reproducibility has been reported to be high [23]. Axial CT scans of bilateral glenohumeral joints were taken and reconstructed with 1-mm-thick slices (Aquilion ONE, Canon Medical Systems Corp, Tochigi, Japan). Using CT Digital Imaging and Communications in Medicine (DICOM) data, 3-dimensional surface models of bilateral glenoids and proximal humeri were reconstructed using the AVIZO 6.2 software (Maxnet, Tokyo, Japan). In shoulders with a bony Bankart lesion [24, 25], the lesion was disregarded in model reconstruction of the glenoid. The left surface models were mirrored horizontally using the MeshLab 1.3.3 software (ISTI, Pisa, Italy), and intact bony areas were matched to those of the right models using an iterative closest point matching program in Visual Tool Kit 5.10.0 (Kitware, Clifton Park, NY, USA). On glenoid analysis, the posterior half of the glenoid surface was selected for surface matching because the posterior portion was supposed to remain intact (Fig. 1). On humeral head analysis, the anterior half of the humeral head including the lesser tuberosity and bicipital groove was used to match the surface data (Fig. 2). To minimize the effects of side-to-side differences of the bones, an area with a thickness less than 1 mm was determined by measuring the Hausdorff distance between the two sides, and the area was removed from the bone defect area in the analyses.

The volumes of bone defects in the humeral head and glenoid of the affected shoulder were then assessed using the RapidForm XOR3 3.0.3.1 software (Geomagic, Morrisville, NC, USA). Since the critical size of bone defects is thought to be affected by the individual size of the patients, the values of bone defect volume were divided by the cube of the patient’s height, which is related to the size of the glenoid and the humeral head [26], before statistical analyses.

### Statistical analysis

Statistical analyses were performed using the IBM SPSS Statistics 25.0.0.0 software (IBM, Armonk, NY, USA). As possible explanatory variables for bone defects in cases with glenohumeral instability, sex, shoulder dominance, presence of bony Bankart lesions on CT scans, presence of anterior and inferior hyperlaxity, involvement in collision sports, age at the time of CT scans, age at the time of initial trauma, duration of symptoms, the corrected values of bone defect volumes in the humeral head and glenoid, number of total instability episodes, and number of self-irreducible dislocations were evaluated. The patients were considered to have anterior hyperlaxity when the contralateral shoulder had external rotation greater than 85° with the arm at the side, and inferior hyperlaxity was defined as a positive hyperabduction test [27] in which a side-to-side difference greater than 20° is positive [28]. Patients who played rugby, American football, boxing, or Judo and had had a traumatic instability episode with the sport were considered to be involved in collision sports. Symptom duration was defined as the average time from the first episode to CT scanning. The number of obvious instability episodes at the time of CT scanning was evaluated from the patients’ personal statements. Instability episodes that could not be reduced by the patients themselves were also evaluated as the number of self-irreducible dislocations. Since the number of spontaneous or self-reducible traumatic episodes was close to that of the number of total instability episodes in most cases, the number of self-reducible subluxations was not used as a variable in the present study to yield appropriate statistical results.

The revised values of glenoid defect volumes and of humeral head defect volume, the number of total instability episodes, and the number of self-irreducible dislocations were selected as objective variables for statistical analyses. Differences in bone defect volumes and episode numbers by categorical explanatory variables including sex, shoulder dominance, presence of bony Bankart lesions, presence of anterior and inferior hyperlaxity, and involvement in collision sports were each evaluated using Mann-Whitney *U* tests. For quantitative explanatory variables including age at CT scans, age at initial trauma, duration of symptoms, the corrected values of bone defect volumes in the humeral head and the glenoid, and number of total instability episodes and number of self-irreducible dislocations, and correlations between objective variables and explanatory variables were each evaluated using Pearson correlation coefficients.

The factors related to large bone defects and a high number of instability episodes were then evaluated. Since the threshold of bone defect volume and the number of instability episodes has not yet been determined, this study evaluated factors affecting greater bone defects and higher numbers of episodes greater than the 75th percentiles of our patients [29]. Including significant variables on bivariate analyses, factors related to large glenoid and humeral head bone defects and high numbers of total instability episodes and of self-irreducible dislocation episodes greater than the respective 75th percentiles were each evaluated using multiple logistic regression analyses with forced entry methods to identify which characteristics were independently associated with enlargement of bone defects and increasing number of instability episodes. Results are reported as odds ratios (ORs) with 95% confidence intervals (CIs). Hosmer-Lemeshow tests were used to assess model calibration. The significance level was set at 0.05 for all analyses.

## Results

### Patients’ characteristics

On CT scans, 44 patients (37%) showed bony Bankart lesions. Eighteen patients (15%) were determined to have anterior hyperlaxity, 45 patients (38%) had inferior hyperlaxity, and 27 patients (23%) were involved in collision sports. Glenoid bone defects were found in 116 patients (97%), with mean volume of 292.8 ± 244.0 mm^3^ (range, 0–1018.1 mm^3^), whereas humeral head bone defects were found in 116 patients (97%), with mean volume of 435.1 ± 396.9 mm^3^ (range, 0–1775.1 mm^3^). The mean number of total instability episodes was 19.1 ± 25.1 times (range, 1–100 times), and the mean number of self-irreducible dislocations was 2.3 ± 3.4 times (range, 0–20 times). The patients’ characteristics are summarized in Tables 1 and 2.

**Table 1:** Bivariate analysis: differences in categorical explanatory variables

Parameter		Number (%)	Glenoid defect (mm^3^)	Humeral head defect (mm^3^)	Number of total instability episodes (times)	Number of self-irreducible dislocations (times)
Sex	Male	96 (80%)	323.9 ± 154.6	491.0 ± 419.5	17.1 ± 23.1	2.0 ± 3.1
	Female	24 (20%)	168.0 ± 154.6	211.5 ± 151.6	27.3 ± 31.1	3.2 ± 4.3
	*P* value	-	.046*	.034*	.051	.085
Shoulder dominance	Dominant	73 (61%)	262.6 ± 243.9	358.8 ± 311.1	18.4 ± 24.2	2.0 ± 2.9
	Nondominant	47 (39%)	339.5 ± 239.2	553.7 ± 482.1	20.3 ± 26.7	2.7 ±3.9
	*P* value	-	.043*	.039*	.323	.818
Bony Bankart	+	44 (37%)	336.2 ± 238.4	423.6 ± 416.5	22.0 ± 27.9	1.9 ± 3.7
	−	76 (63%)	267.6 ± 245.2	441.8 ± 387.8	17.4 ± 23.3	2.5 ± 3.2
	*P* value	-	.121	.439	.432	.028*
Anterior hyperlaxity	+	18 (15%)	297.6 ± 249.9	366.2 ± 279.2	23.9 ± 30.6	2.1 ± 2.2
	−	102 (85%)	265.0 ± 211.4	447.3 ± 414.1	18.3 ± 24.1	2.3 ± 3.5
	*P* value	-	.968	.997	.591	.733
Inferior hyperlaxity	+	45 (38%)	255.4 ± 216.4	335.7 ± 276.1	22.7 ± 28.0	2.3 ± 2.5
	−	75 (63%)	315.2 ± 257.9	494.8 ± 445.4	17.0 ± 23.1	2.3 ± 3.8
	*P* value	-	.378	.193	.356	.167
Collision sports	+	27 (23%)	356.0 ± 235.9	436.0 ± 373.0	21.8 ± 27.5	2.6 ± 3.2
	−	93 (77%)	274.4 ± 244.4	434.9 ± 405.5	18.3 ± 24.4	2.2 ± 3.4
	*P* value	-	.086	.935	.818	.598

Values are given as means and standard deviation. Statistical analyses of bone defect volumes were performed after correction of the values of bone defect volumes by the patient’s height

* *P* < .05

**Table 2:** Bivariate analysis: correlation with quantitative explanatory variables

Parameter	Mean ± SD	Correlation coefficient (*P* value)			
		Glenoid defect (mm^3^)	Humeral head defect (mm^3^)	Number of total instability episodes (times)	Number of self-irreducible dislocations (times)
Age at CT scans (years)	26.1 ± 10.4	0.074 (*P* = .419)	0.215* (*P* = .018)	0.224* (*P* = .014)	0.297** (*P* = .001)
Age at initial trauma (years)	19.7 ± 5.5	0.041 (*P* =.660)	0.221* (*P* = .015)	−0.097 (*P* = .290)	0.026 (*P* = .781)
Duration of symptoms (years)	6.4 ± 9.1	0.061 (*P* = .509)	0.123 (*P* = .180)	0.318*** (*P* < .001)	0.322*** (*P* < .001)
Glenoid defect (mm^3^)	292.8 ± 244.0	-	0.413*** (*P* < .001)	0.354*** (*P* < .001)	0.131 (*P* = .153)
Humeral head defect (mm^3^)	435.1 ± 396.9	0.413*** (*P* < .001)	-	0.097 (*P* = .291)	0.306** (*P* = .001)
Number of total instability episodes (times)	19.1 ± 25.1	0.354*** (*P* < .001)	0.097 (*P* = .291)	-	0.011 (*P* = .906)
Number of self-irreducible dislocations (times)	2.3 ± 3.4	0.131 (*P* = .153)	0.306** (*P* = .001)	0.011 (*P* = .906)	-

Values are given as means and standard deviation. Statistical analyses of bone defect volumes were performed after correction of the values of bone defect volumes by the patient’s height

*SD* standard deviation

*P < .05; **P < .01; ***P < .001

### Bone defects

**Fig. 3:**
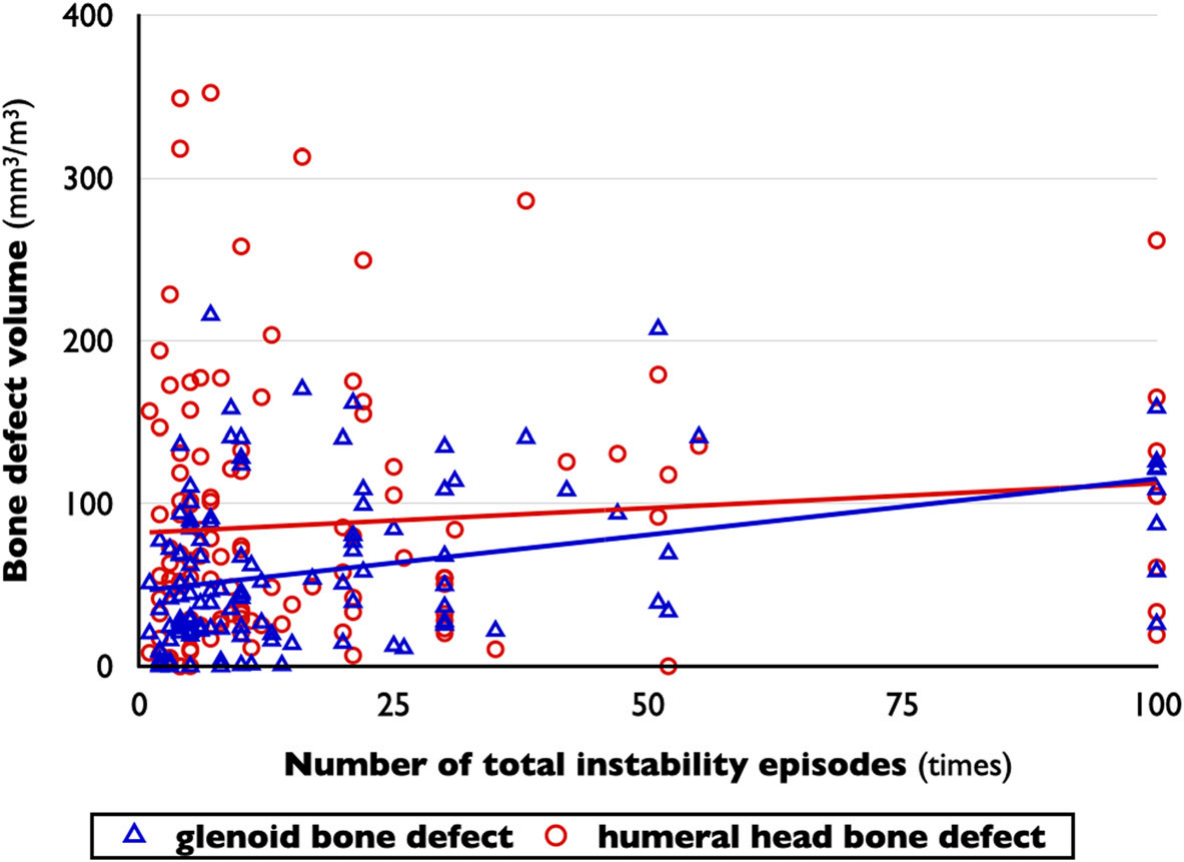
Correlations between the number of total instability episodes and bone defect volumes. The number of total instability episodes has a positive correlation with glenoid defect volume (*R* = 0.354, *P* < .001), but not with humeral head defect volume (*R* = 0.097, *P* = .291). Bone defect volume was corrected by the patient’s height

**Fig. 4:**
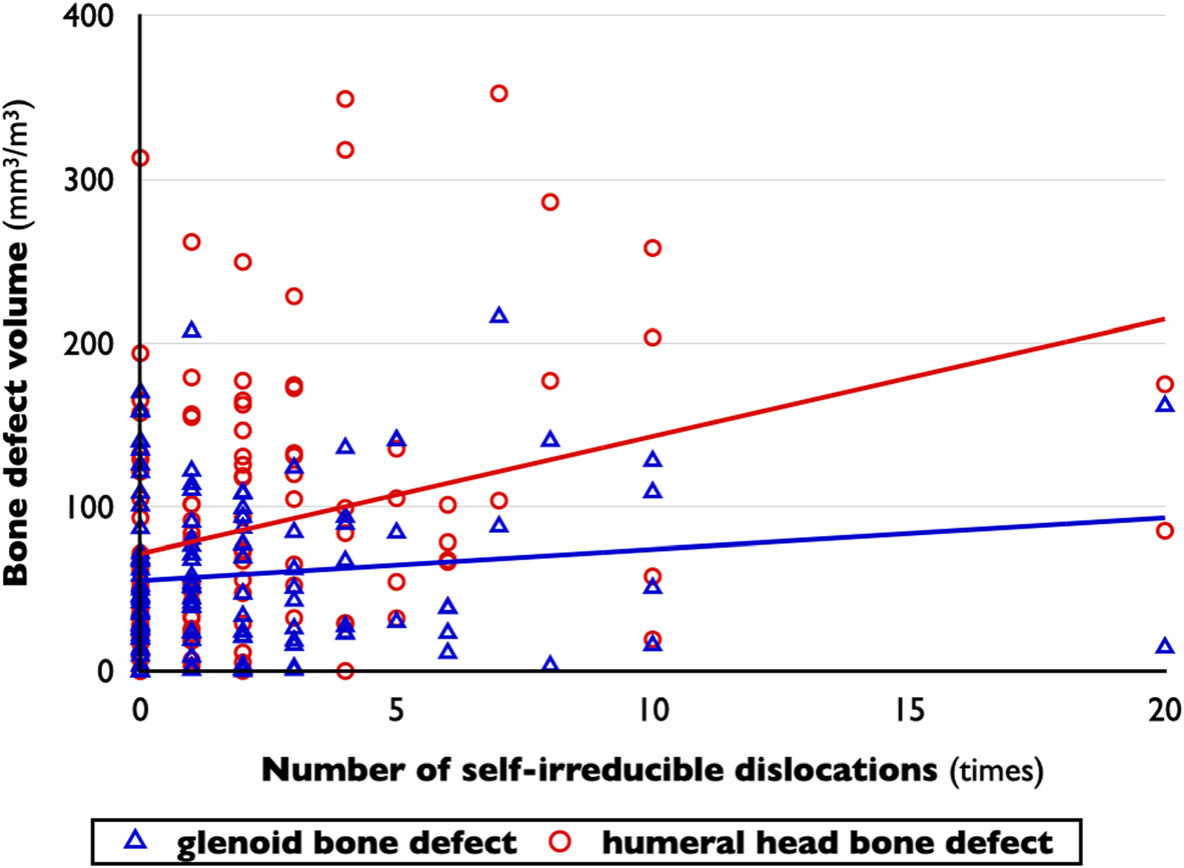
Correlations between the number of self-irreducible dislocations and bone defect volumes. The number of self-irreducible dislocations has a positive correlation with humeral head defect volume (*R* = 0.306, *P* = .001), but not with glenoid defect volume (*R* = 0.131, *P* = .153). Bone defect volume was corrected by the patient’s height

**Fig. 5:**
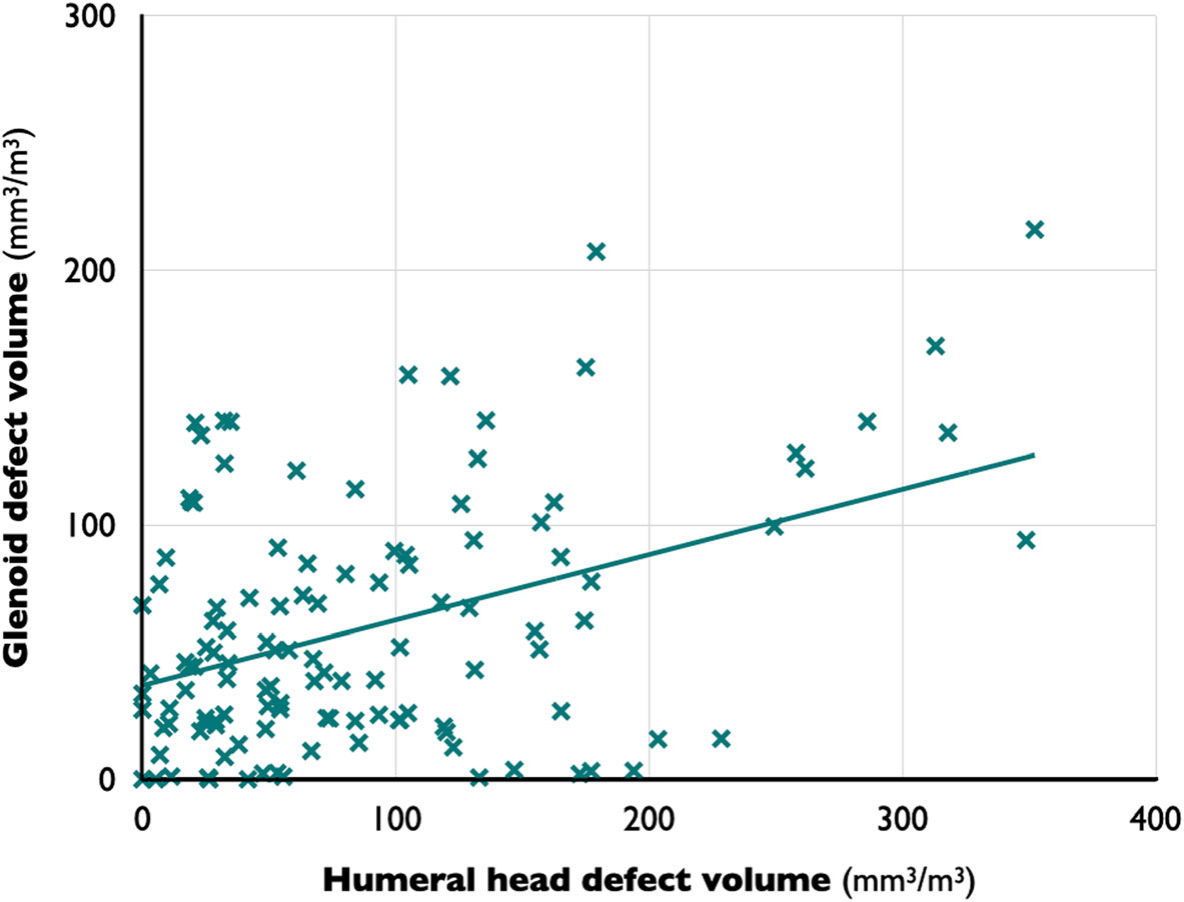
Correlations between humeral head defect volume and glenoid defect volume. The bone defect volumes of bipolar lesions show a positive correlation (*R* = 0.413, *P* < .001). Bone defect volume was corrected by the patient’s height

In glenoid defects, male sex (*P* = .046), nondominant shoulder (*P* = .043), larger humeral head defects (*R* = 0.413, *P* < .001), and higher number of total instability episodes (*R* = 0.354, *P* < .001) were found to be possible variables on bivariate analyses (Tables 1 and 2 and Figs. 3, 4, and 5). The 75th percentile value of glenoid bone defects divided by the cube of the patient’s height was 89.7 mm^3^/m^3^. The glenoids with 75th percentiles of glenoid defects had a 23.2% defect of glenoid width in the present study. On logistic regression analysis, larger humeral head defects (OR, 1.011 per 1-mm^3^/m^3^ increase; *P* < .001) and higher number of total instability episodes (OR, 1.033 per 1-time increase; *P* = .001) were factors related to larger glenoid defects. The model was well calibrated (*P* = .630 on the Hosmer-Lemeshow test) (Table 3).

**Table 3:** Multivariate regression for large bone defects in the glenoid and humeral head

	Parameter	Odds ratio	95% CI		*P* value
			Lower	Upper	
Large glenoid defect ^a^	Male sex	3.391	0.627	18.326	.156
	Nondominant shoulder	1.223	0.452	3.310	.692
	Humeral head defect (mm^3^/m^3^)	1.011	1.004	1.017	< .001***
	Number of total instability episodes (times)	1.033	1.013	1.053	.001**
Large humeral head defect ^b^	Male sex	33.180	2.051	536.893	.014*
	Nondominant shoulder	1.127	0.426	2.982	.810
	Age at CT scans (years)	1.053	0.982	1.129	.146
	Age at initial trauma (years)	1.025	0.924	1.136	.643
	Glenoid defect (mm^3^/m^3^)	1.012	1.002	1.022	.015*
	Number of self-irreducible dislocations (times)	1.197	1.021	1.404	.027*

^a^Defined as the glenoid defect corrected by the patient’s height ≥ 89.7 mm^3^/m^3^ (75th percentile)

^b^Defined as the humeral head defect corrected by the patient’s height ≥ 129.0 mm^3^/m^3^ (75th percentile)

*P < .05; **P < .01; ***P < .001

For humeral head defects, male sex (*P* = .034), nondominant shoulder (*P* = .039), higher age (*R* = 0.215, *P* = .018), higher age at the time of initial trauma (*R* = 0.221, *P* = .015), larger glenoid defects (*R* = 0.413, *P* < .001), and higher number of self-irreducible dislocation episodes (*R* = 0.306, *P* = .001) were found to be possible variables (Tables 1 and 2 and Figs. 3, 4, and 5). The 75th percentile value of glenoid bone defects divided by the cube of the patient’s height was 129.0 mm^3^/m^3^. On logistic regression analysis, male sex (OR, 33.180; *P* = .014), larger glenoid defects (OR, 1.012 per 1-mm^3^/m^3^ increase; *P* = .015), and larger number of self-irreducible dislocations (OR, 1.197 per 1-time increase; *P* = .027) were related to large Hill-Sachs lesions. The model was well calibrated (*P* = .324 on the Hosmer-Lemeshow test) (Table 3).

### Instability episodes

For the number of total instability episodes, higher age (*R* = 0.224, *P* = .014), longer duration of symptoms (*R* = 0.318, *P* < .001), and larger glenoid defect volume (*R* = 0.354, *P* < .001) were found to be possible variables on bivariate analyses (Tables 1 and 2 and Figs. 3, 4, and 5). The 75th percentile value of the number of total instability episodes was 22 times. On logistic regression, longer duration of symptoms (OR, 1.225 per 1-year increase; *P* = .003) and larger glenoid bone defects (OR, 1.015 per 1-mm^3^/m^3^ increase; *P* = .002) were factors related to an increased number of total instability episodes. The model was well calibrated (*P* = .281 on the Hosmer-Lemeshow test) (Table 4).

**Table 4:** Multivariate regression for a high number of traumatic episodes

	Parameter	Odds ratio	95% CI		*P* value
			Lower	Upper	
High number of total instability episodes ^a^	Age at CT scans (years)	0.890	0.791	1.001	.051
	Duration of symptoms (years)	1.225	1.071	1.402	.003**
	Glenoid defect (mm^3^/m^3^)	1.015	1.006	1.024	.002**
High number of self-irreducible dislocations ^b^	Absence of bony Bankart lesion	0.963	0.338	2.748	.944
	Age at CT scans (years)	0.975	0.885	1.074	.612
	Duration of symptoms (years)	1.106	0.992	1.233	.068
	Humeral head defect (mm^3^/m^3^)	1.008	1.002	1.014	.007**

^a^Defined as the number of total instability episodes ≥ 22 times (75th percentile)

^b^Defined as the number of self-irreducible dislocations ≥ 4 times (75th percentile)

**P < .01

For the number of self-irreducible dislocation episodes, absence of bony Bankart lesions (*P* = .028), higher age at CT scanning (*R* = 0.297, *P* = .001), longer duration of symptoms (*R* = 0.322, *P* < .001), and larger humeral head defect volume (*R* = 0.306, *P* = .001) were found to be possible variables on bivariate analyses (Table 1 and 2 and Figs. 3, 4, and 5). The 75th percentile value of the number of self-irreducible dislocation episodes was 4 times. On logistic regression, only the presence of larger humeral head bone defects (OR, 1.008 per 1-mm^3^/m^3^ increase; *P* = .007) was a factor related to an increased number of self-irreducible dislocation episodes. The model was well calibrated (*P* = .309 on the Hosmer-Lemeshow test) (Table 4).

## Discussion

The present study evaluated factors related to large bone defects and an increased number of instability episodes in cases with glenohumeral instability. Whereas the present study showed that bipolar lesions affect the amounts of bone defects reciprocally, factors related to large bone defects differed between the glenoid and humeral head. Glenoid defects were related to the number of total instability episodes, whereas humeral head defects were related to the number of self-irreducible dislocation episodes. The present results would be useful to predict the prognosis of patients with anterior glenohumeral instability and may support early surgical intervention for unstable shoulders.

Recently, glenoid defects and Hill-Sachs lesions have been recognized as bipolar lesions [12]. The present results suggest that bipolar lesions affect the enlargement of bone defects of unstable shoulders reciprocally. Similar to the present results, glenoid defects and Hill-Sachs lesions were reported to have a significant, but not strong, correlation [21, 22]. Defects of articular cartilage and bared bone of the glenoid or the humeral head are likely to injure the other side of the joint. The present results indicated that reciprocal enlargement of bone defects could lead to poor clinical outcomes in cases with glenohumeral instability, and avoidance of instability episodes can preserve the bone stock of the glenohumeral joint.

Significant glenoid bone defects are known to be a major negative factor for recurrence after stabilization surgery for the treatment of glenohumeral instability [3–6]. The number of instability episodes has been reported to have a strong effect on the size of glenoid defects [15, 16, 20, 21, 30], and the present results were consistent with past studies. On the other hand, a larger glenoid bone defect was a factor related to an increased number of total instability episodes. Repetitive instability episodes could shave the glenoid edge and enlarge the glenoid defects, whereas the glenohumeral joint became unstable with an increased number of instability episodes in cases with a glenoid with large bone loss. Glenoid defects are thought to be both a cause and a result of unstable shoulder. The presence of bony Bankart lesions, which indicate fracture of the glenoid rim with instability episodes and can lead to a significant bone defect of the glenoid [25], was not found to be significant in the present study. The bone fragments are usually absorbed with time [20], and it is often difficult to distinguish an absorbed fragment from an erosion [25]. This fact might have affected the present results.

Although the Hill-Sachs lesion is a well-known bone lesion in patients with glenohumeral instability [10], few studies [13, 18] have focused on the size of the lesion. The present study showed that factors related to large humeral head bone defects were male sex, large glenoid defects, and higher number of irreducible dislocation episodes. Compared with women, men are generally more active and likely to experience more severe instability episodes. Although the severity of each instability episode could not be assessed, the present results implied that large humeral head bone defects can be created after severe self-irreducible dislocations. Ozaki et al. [18] reported that the prevalence of Hill-Sachs lesions increased significantly as the number of dislocations increased and that the lesions were enlarged significantly by recurrent dislocations. The present results were consistent with them. The number of self-irreducible dislocation episodes is a factor related to large humeral head bone defects, whereas the presence of large humeral head bone defects was the only factor related to an increased number of self-irreducible dislocation episodes in the present study. Large humeral head bone defects could be both a cause and a result of self-irreducible dislocation episodes. The glenohumeral joint is likely to be locked in the shoulder with a large Hill-Sachs lesion when the Hill-Sachs lesion is engaged to the glenoid rim.

Increased instability episodes clearly impair the quality of life and activities of the patient. The present study showed that an increased number of total instability episodes is related to large glenoid defects and that an increased number of self-irreducible dislocations is related to large humeral head defects. In addition to large bone defects, an increased number of instability episodes is known to cause secondary glenohumeral arthritis after both conservative treatment [31] and surgical stabilization [32]. The current results may support early surgical intervention for unstable shoulders. With the proposal of the glenoid track concept [11], the extent of Hill-Sachs lesions is now widely recognized as a negative factor for recurrence after stabilizing surgeries [4, 12, 13]. Although the difference in episode type has been rarely discussed [14, 16, 18, 19], it is fairly different for patients whether they can or cannot reduce traumatic instability events by themselves. The present results showed that a large humeral head bone defect is also a factor related to self-irreducible dislocations. Additional procedures for the Hill-Sachs lesion, such as remplissage [33] or bone grafting [34], might have an effect on preventing not only engagement of the bipolar lesions, but also complete dislocations that cannot be reduced by themselves.

This study had several limitations. The first limitation was the retrospective design of the study. CT scans of the patients with symptomatic unilateral glenohumeral instability at the time of surgical intervention were evaluated, but the creation and enlargement of bone defects of each patient were not observed. Furthermore, the number was determined based on the patients’ personal statements, but patients’ memories are not always accurate [21]. Second, unknown variables might exist in addition to the factors assessed in this study. The present results indicated that bipolar lesions affected enlargement of bone defects reciprocally, but unmeasurable factors including the strength of each patient’s bone, intensity of each trauma, duration at the dislocated position, and violent reduction of dislocation might affect the extent of bone defects. The third limitation was that the thresholds of bone defect size and the number of instability episodes have not yet been determined. Menendez et al. [29] evaluated predictors of severe postoperative pain after total shoulder arthroplasty, which they defined a priori as peak pain intensity ≥75th percentile in their patients. Similarly, this study evaluated factors related to greater bone defects and higher numbers of episodes greater than the 75th percentiles of our patients. The glenoid with the 75th percentile of glenoid defect in the present study had a 23.2% defect of its width, and the 75th percentile of the number of total instability episodes was 22 times in the present study. These values appeared to be valid, but it remains unclear whether our materials represented typical glenohumeral instability or not, and further study will be needed to clarify the clinical threshold for critical bone defect and of glenohumeral instability. Fourth, side-to-side differences might exist in the glenohumeral joint even though both shoulders are reported to be highly symmetrical in shape and size [21, 25, 35]. Finally, proportion, position, and orientation of bone defects, which also could affect clinical symptoms [11, 13, 36], were not taken into account. The length, width, and depth of bone defects could be evaluated [23]. However, since two-dimensional measurement in three-dimensional analysis would lead to another limitation in determining the axes of the bone defects and which two-dimensional parameters are clinically important remains unclear, this study evaluated bone defect volume three-dimensionally.

## Conclusion

Whereas this study showed that bipolar lesions affect the amount of bone defects reciprocally, factors related to greater bone defects differed between the glenoid and the humeral head. Glenoid defects were related to the number of total instability episodes, whereas humeral head defects were related to the number of self-irreducible dislocations.

## Data Availability

The datasets used and/or analyzed during the current study are available from the corresponding author on reasonable request.

## Abbreviations

CT: Computed tomography
DICOM: Digital Imaging and Communications in Medicine
